# Modelling the Impact of Argon Atoms on a WO_3_ Surface by Molecular Dynamics Simulations

**DOI:** 10.3390/molecules29245928

**Published:** 2024-12-16

**Authors:** Shokirbek Shermukhamedov, Thana Maihom, Michael Probst

**Affiliations:** 1 Institute of Ion Physics and Applied Physics, University of Innsbruck, Technikerstraße 25, 6020 Innsbruck, Austria; 2 Department of Chemistry, Ångström Laboratory, Uppsala University, 751 21 Uppsala, Sweden; 3 Division of Chemistry, Department of Physical and Material Sciences, Faculty of Liberal Arts and Science, Kasetsart University, Nakhon Pathom 73140, Thailand; thana.m@ku.th; 4 School of Energy Science and Engineering, Vidyasirimedhi Institute of Science and Technology, Rayong 21201, Thailand

**Keywords:** molecular dynamics, surface sputtering, tungsten oxide, fusion research, machine learning potential energy functions, Behler–Parinello method

## Abstract

Machine learning potential energy functions can drive the atomistic dynamics of molecules, clusters, and condensed phases. They are amongst the first examples that showed how quantum mechanics together with machine learning can predict chemical reactions as well as material properties and even lead to new materials. In this work, we study the behaviour of tungsten trioxide (WO_3_) surfaces upon particle impact by employing potential energy surfaces represented by neural networks. Besides being omnipresent on tungsten surfaces exposed to air, WO_3_ plays an important role in nuclear fusion experiments due to the preferred use of tungsten for plasma-facing components. In this instance, the formation of WO_3_ is caused by the omnipresent traces of oxygen. WO_3_ becomes a plasma-facing material, but its properties, especially concerning degradation, have hardly been studied. We employ molecular dynamics simulations to investigate sputtering, reflection, and adsorption phenomena occurring on WO_3_ surfaces irradiated with Argon. The machine-learned potential energy function underlying the MD simulations is modelled using a neural network (NNP) trained from large sets of density functional theory calculations by means of the Behler–Parrinello method. The analysis focuses on sputtering yields for both oxygen and tungsten (W), for various incident energies and impact angles. An increase in Ar incident energy increases the sputtering yield of oxygen, with distinct features observed in different energy ranges. The sputtering yields of tungsten remain exceedingly low, even compared to pristine W surfaces. The ratios between the reflection, adsorption, and retention of the Ar atoms have been analyzed on their dependence of impact energy and incident end angles. We find that the energy spectrum of sputtered oxygen atoms follows a lognormal distribution and offers information about surface binding energies on the WO_3_ surface.

## 1. Introduction

Understanding the interaction between plasma particles and plasma-facing materials (PFMs) is paramount to several emergent technologies, such as plasma applications in medicine. It is especially important in materials science, where plasmas are used for sur-face treatment and, probably most prominently, in fusion energy research. The aim is to unlock the potential of nuclear fusion as a clean, abundant, and sustainable energy source for the future. Within this field, understanding the interaction between plasma particles and PFMs is paramount to the successful development and operation of fusion reactors, particularly in ITER [1,2]. Tungsten plays a crucial role in managing the intense heat and particle fluxes generated in the divertor region within fusion devices [3] and is likely to also be present in other regions of the vessel. It tends to form oxides, of which WO_3_ is thermodynamically the most stable one. Due to the omnipresence of traces of oxygen, or even deliberate exposure to it, tungsten oxides have recently garnered significant interest in fusion-related materials research [4,5,6,7,8].

In the effort to advance our comprehension of plasma–PFM interactions, molecular dynamics simulations emerge as powerful tools offering detailed insights into atomic-scale processes. They can explore the complex dynamics of plasma particles bombarding PFM surfaces, shedding light on phenomena such as sputtering, reflection, and retention [9,10].

Previous research efforts have investigated the sputtering dynamics of tungsten surfaces under plasma exposure, particularly concerning the bombardment of D/T, Be, and Ar atoms. They have demonstrated the efficacy of MD simulations in capturing the intricate interplay between incident particles and W surfaces, offering valuable insights into material erosion and modification processes [11,12,13]. Similar information for oxidized tungsten surfaces is still lacking. Interest in investigating tungsten oxides also arises from a phenomenon observed in many experiments, the depletion of the surface layer through oxygen loss leading to a reduction to lower oxidation states [6,14]. In some instances, transition metal oxides are even reduced to the metallic state under plasma particle bombardment, including the fusion-relevant materials WO_2_ and Fe_2_O_3_ [7].

A crucial aspect of understanding material evaluation under plasma particles involves computational methods. Quantum chemical calculations, especially density functional methods, provide insights into the fundamental behaviour of particles and crystalline materials under extreme conditions. However, their computational complexity significantly restricts the system size and timescale that can be explored. To avoid this, semiempirical Buckingham potentials have been used to model bulk [15,16] and pure surface [17] WO_3_ structures. Alternatively, analytic multi-body bond-order potentials were parameterized to accurately replicate the mechanical properties of bulk WO_3_ [18]. While these approaches provide computational efficiency, their ability to reproduce forces derived from quantum-level calculations is limited and they are unable to fully capture the complex interactions that DFT can model. As a result, until recently, DFT calculations remained the primary method for studying WO_3_ surfaces and the interactions of various molecules and atoms with it [19,20,21]. High-dimensional neural network potentials (HDNNPs) have emerged as a promising approach to bridge the gap between quantum-level accuracy and classical molecular dynamics. HDNNPs leverage machine learning techniques to approximate the complex potential energy surfaces governing atomic interactions with high accuracy and efficiency. By capturing the intricacies of atomic interactions at the plasma–PFM boundary, they enable the simulation and analysis of phenomena such as sputtering, reflection, adsorption, and chemical reactions in atomistic detail. HDNNPs have previously been used to approximate the potential energy surface of Be-D (H,T) [22], W-Ar [11], and Be2W-D [23] systems. The obtained sputtering yields agree with experimental and theoretically predicted values where latter are available.

In the present study, we utilized molecular dynamics modelling with high-dimensional neural network potential energy functions to explore the bombardment of clean WO_3_ (001) surfaces by argon atoms. Through a series of simulations, we elucidate the mechanisms driving the sputtering dynamics, with a particular focus on the effects of incident particle energy, sputtering and reflection dynamics, and surface binding energy.

## 2. Methods

### 2.1. Machine Learning Potential Energy Functions Based on Neural Networks

In high-dimensional neural network potential energy functions (HDNNP, [24]), the total energy of a configuration is obtained as a sum of atomic energies E_i_, which are obtained from an atom-centred element-specific neural network. A cut-off radius of 7 Å defines the atomic environment. The cartesian coordinates of the neighbour atoms are converted into parameters of weighted radial and angular Behler–Parinello-type symmetry functions [25], which reproduce the local environment with physically correct invariances. The parameters of the grids used for the symmetry functions are detailed in Section S1 of the Supplementary Materials. They serve as input to a feedforward neural network with two hidden layers of 25 nodes each. Each node consists of a soft-plus activation function with an offset. After the NNP is created and trained, MD simulations are performed with a modified LAMMPS code [26].

### 2.2. Network Training

#### 2.2.1. Density Functional Calculations

Energies and forces from DFT calculations as a function of atomic coordinates serve as training and test data to determine the NNP parameters, both in the initial and refinement steps. One can view the NNP as a ‘bridge’ to generate DFT-quality forces that drive the MD simulation in a much faster way than would be possible otherwise.

The atomic coordinates mentioned above needed for the initial, subsequent training, and test sets are generated from ab initio MD trajectories. Specifically, the initial set of training structures, energies, and forces was obtained from sputtering trajectories with different incident angles of Ar on small WO_3_ (001) supercells. During MD simulations, the lowest layer of atoms in the slab was fixed, while all other atoms were allowed to relax. The convergence threshold for the forces was 10^−4^ eV/Å. Refs. [27,28] have been used. In these ab initio MD calculations, the forces were obtained via Kohn–Sham DFT with the PW91 functional [29] at the generalized gradient approximation (GGA) level, as implemented in the Vienna Ab Initio Simulation Package (VASP) [30,31]. Technically, a rather large supercell with a Gamma-centred k-point mesh of 3 × 3 × 3 was employed, and the Kohn–Sham orbitals were expanded in a periodic plane wave basis set. The calculations were spin-unpolarized, with a cut-off energy of 350 eV [32]. GGA-PAW simulations with similar parameters have been used before for studying the interaction of noble gas atoms with tungsten [33].

#### 2.2.2. Generation of Training Data and Network Training

We used an iterative refinement protocol as described in Ref. [32] to successively improve the training by supplying more datasets. To develop a robust model, we supplemented the initial small supercell slab with the composition W_27_O_81_Ar and dimensions x = 11.41 Å, y = 11.41 Å, z = 19.42 Å, with three additional systems.

The first additional system features a similar (001) surface but with an increased z-dimension of z = 24.12 Å. The second system, also a (001) surface, had a larger slab size with the sum formula W_48_O_144_Ar and dimensions x = 15.31 Å, y = 15.31 Å, z = 24.12 Å. The third system represents bulk WO_3_ and contains the same number of atoms as the smaller surface systems. We found that including bulk configurations is crucial for improving the accuracy of atomic energy fitting.

**Figure 1:**
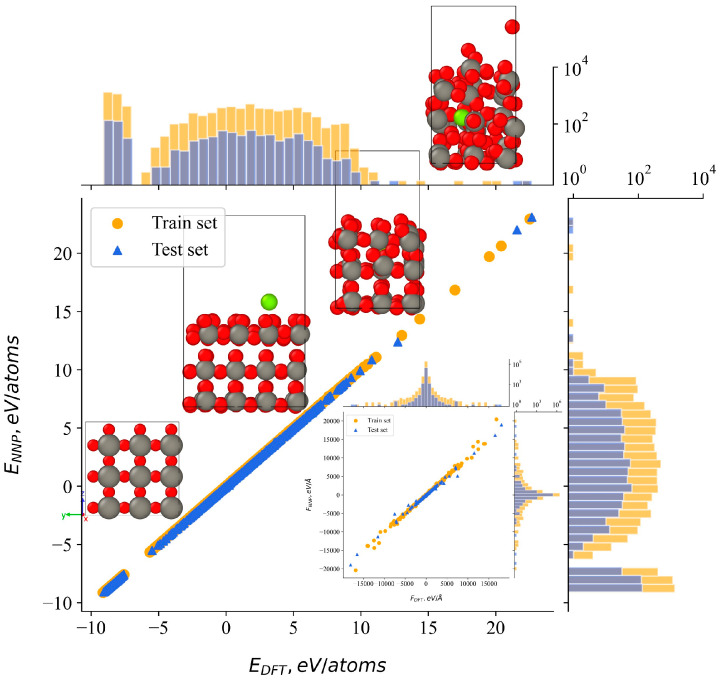
Correlation between DFT-calculated and NNP-predicted atomic energies, accompanied by two-dimensional histograms showing the distribution of these values. The subplot illustrates the correlation between reference and predicted forces using the same approach. Representative examples of structures from the training data are presented across the energy correlation points.

The larger surface was used for data collection involving high energy argon atoms, and its combination with the two surfaces of varying heights helped mitigate surface effects in fully periodic DFT calculations. After five refinement steps, the training dataset covers a broad range of potential energies. This is demonstrated in Figure 1 for a diverse distribution across different energy ranges. To show the four structural types defined above, four representative examples were selected across the range of energies, corresponding to the axis values.

The lower-right subplot in Figure 1 illustrates the correlation between the forces predicted by the neural network potential and those derived from DFT. Overall, the correlation between NNP and DFT energies, as well as for the atomic forces, is highly satisfactory, demonstrating a strong linear relationship between the predicted and reference values. Variations are observed in the maximum and minimum force values, potentially attributable to structures characterized by closely spaced atoms.

Overall, the final reference dataset included 8766 configurations, in total 981,730 atomic energies and 2,945,190 force components. To train the final NNP, 90% of the configurations were randomly selected for the training set, while the remaining 10% were used as a test set to validate the accuracy of the NNP energy predictions. Following 60 training steps, the mean absolute error (MAE) in the test set was 0.54 meV/atom for energies and 1.44 eV/Å for atomic forces. Conversely, lower MAE values were observed in the training set, measuring 0.32 meV/atom for energies and 1.36 eV/Å for atomic forces. Notably, the training process did not exhibit signs of overfitting.

To validate the final NNP, additional test calculations were conducted at both the DFT and NNP levels. Various structures and properties were examined, with detailed information provided in Section S2 of the Supplementary Materials. The calculated lattice parameter of bulk WO_3_ falls within the range of previously reported values in the literature, demonstrating the accuracy acceptable for bulk properties. Furthermore, the work functions for removing surface oxygen and tungsten atoms, which were not explicitly included in the training set, were also evaluated. These calculations revealed low errors in the predicted forces, aligning with the primary objective of this study to achieve high accuracy in force predictions.

### 2.3. Details of the Molecular Dynamics Simulations

The larger simulation box used for the production sputtering simulations contained 150 tungsten atoms and 450 oxygen atoms. Its dimensions were x = 19.15 Å, y = 19.15 Å, and z = 45.2 Å. It was relaxed in 300 K of the sample in the canonical (NVT) ensemble for 0.1 ps. The subsequent sputtering simulations were performed in the microcanonical (NVE) ensemble. The timestep was dynamically adjusted between 0.001 and 1 fs, depending on the initial velocity of the Ar projectile. The simulation continues until one of the stopping conditions is met. These conditions involve, for example, the exit of a sputtered particle or reaching a threshold of timesteps. Simulations were conducted at various energies (20, 30, 40, 50, 60, 80, 100, 150, 200, 300, 500, and 800 eV) and impact angles (0°, 20°, 40°, and 60°), totaling 2000 non-cumulative irradiations.

## 3. Results

### 3.1. Sputtering Yields

**Figure 2:**
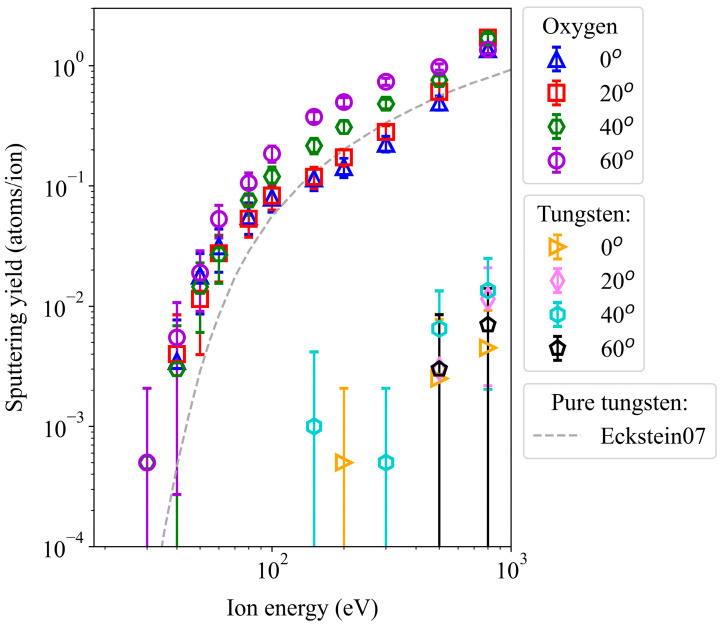
Sputtering yields of oxygen and tungsten atoms from a WO_3_ surface irradiated by non-cumulative Ar impacts at various incident energies and angles. The grey dashed line represents the Eckstein curve for pure tungsten surfaces [34].

Argon atoms are targeted towards a WO_3_ surface at a temperature of 300 K, with varying kinetic energy and impact angles. Figure 2 presents the sputtering yields of oxygen and tungsten atoms under these conditions, showing the ratio of sputtered atoms to total Ar impacts. In the same plot, we also demonstrate the Eckstein fitting curve for experimental yields from pure tungsten surfaces [34]. We observe that oxygen sputtering initiates at an impact energy of 30 eV. However, we only detect one occurrence among 2000 sputtering events, limited to incidence angles of 40° and 60°.

An increase in incident energy always leads to a higher sputtering yield. Particularly within the energy range of 30 to 80 eV, the yield exhibits a steep growth, followed by a gradual rise until the highest impact energy, 800 eV. At about 500 eV, the angular effect levels out. The angular effect is prominent up to approximately 300 eV, where a perpendicular impact (0°) consistently results in the lowest yield or no yield at all. This is interesting since the part of the energy that can be directly transferred to the surface (the z-component of the Ar velocity vector) is largest in the case of 0°.

At high energies, 500 and 800 eV, we also observed W sputtering (Figure 2). At lower energies, this is not statistically significant at all. In the case of W sputtering, the impact angles of 60° led to the largest W sputtering yields. However, their values are still so low that their error bars overlap. These findings indirectly confirm the experimental findings that the surface layers of tungsten oxide samples under irradiation deoxygenated or reduced in oxidation state to pure metal.

In summary, the yields demonstrate the predominant expulsion of oxygen atoms from the surface, effectively retaining the tungsten atoms within the sample and thus preventing contamination of the plasma with W.

### 3.2. Reflection, Adsorption, and Retention of Ar

**Figure 3:**
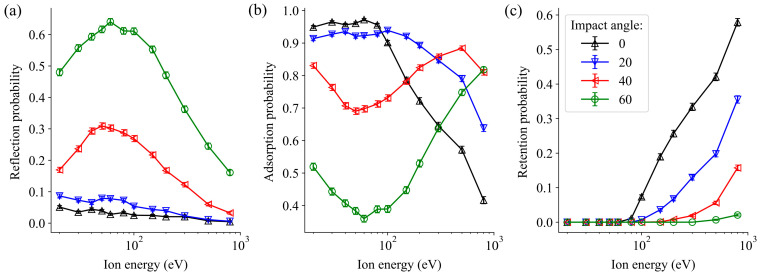
Reflection (**a**), adsorption (**b**), and retention (**c**) probabilities of oxygen atoms caused by the irradiation of a WO_3_ surface by non-cumulative Ar impact at various incident energies and impact angles.

We have performed an analysis of the trajectories following the impact of Ar atoms on the WO_3_ surface. In Figure 3, these trajectories are categorized into three groups, regardless of whether a sputtering event occurs: reflection, adsorption, and retention of Ar. Adsorption refers to surface adsorption, while retention indicates that Ar atoms penetrate below the first layer of the WO_3_ sample.

At lower incident energies, the reflection behaviour (Figure 3a) is strongly dependent on the angle of impact. There is minimal reflection at perpendicular impact, with the highest probability observed at the 60° flattest impact angle, followed by 40°. In both cases, there is a gradual increase up to 80 eV, followed by a reduction in reflection probability.

The adsorption probabilities (Figure 3b) exhibit an opposite trend compared to the reflection values. At 40° and 60° angles, minima are observed where peaks occur in the reflection probabilities. However, at 0° and 20° angles, the adsorption probabilities remain constant and close to 1 in the range from 20 to 100 eV and decrease gradually at higher energies.

The peaks observed in the reflection and adsorption plots can be attributed to inelastic collisions between the incoming argon atoms and surface atoms. At lower (near-perpendicular) angles of incidence, collisions are less frequent, allowing the projectile to penetrate deeper into the surface layer. During these collisions, a portion of the kinetic energy of the incoming particle is transferred into heat or lattice vibrations. However, as the impact angle becomes flatter, the probability of collisions also becomes larger, as the length of the path passed by the Ar atoms per unit height increases compared to the perpendicular case. Consequently, there is greater energy transfer between the projectile and the sample surface at flat impact angles, leading to higher reflection and lower adsorption rates, as observed between 30 and 60 eV. Simultaneously, a decrease in reflection and adsorption is associated with a larger probability of retention, which rises at higher energies and at vertical impact angles, as is shown in Figure 3c.

**Figure 4:**
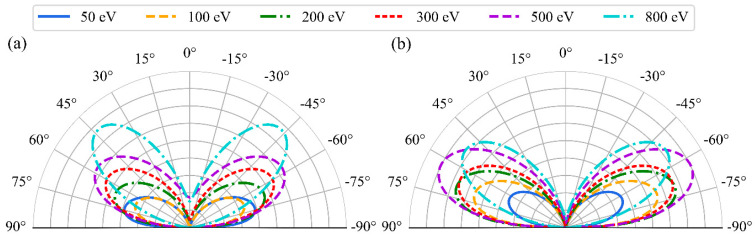
Polar plots of the distribution of yields and angles of sputtered oxygen atoms as a function of the Ar incident energy for the four incident angles; 0° = surface normal (**a**), and 60° (**b**).

From the trajectories, we also calculated the distributions of the outgoing angles of sputtered oxygen atoms. Our results relate to the characteristic angular distribution patterns, commonly referred to as butterfly shape in polar coordinates [35]. To approximate these angular distributions, we used the following expression:

(1) $$f\left(\theta \right)=Acos^{a}\left(\theta \right)-Bcos^{b}\left(\theta \right)$$

where *A*, *B*, *a* and *b* are fitting parameters. The angular distribution pattern and associated parameters change depending on the material, bombarding particle type, and energy. The fitted distributions are showed in Figure 4. In the polar plots, the radial axis represents the surface normal (the z-direction in the simulations), while the distance from the origin corresponds to the sputtering yield. The results are illustrated for six energies and for two angles, 0° and 60°, of the incoming Ar. A noticeable correlation is observed between the incoming and outgoing angles, as well as with the incident energy. At perpendicular impact, the peaks of the distributions shift to the smaller angles with an increasing incoming energy, from 70° at 50 eV to 45° at 800 eV. As the impact angle increases, the distributions become flatter; distributions at 20° and 40° are presented in the SI. At an impact angle of 60°, the peaks of all distributions change only slightly with the incoming angle and are located near 60°. The values of the fitted parameters in Equation (1) are provided in Table 1.

### 3.3. Surface Binding Energy

Besides deriving an analytical formula for the dependence of the sputtering yield on the kinetic energy and incident angle of argon particles, one can also analyze the energy spectra of the sputtered particles in order to predict an effective surface binding energy for the material [36]. The surface binding energy is the main parameter governing material interactions with irradiating particles. It represents the energy required to remove an atom from the surface and is such a key parameter in determining the stability of a material.

**Figure 5:**
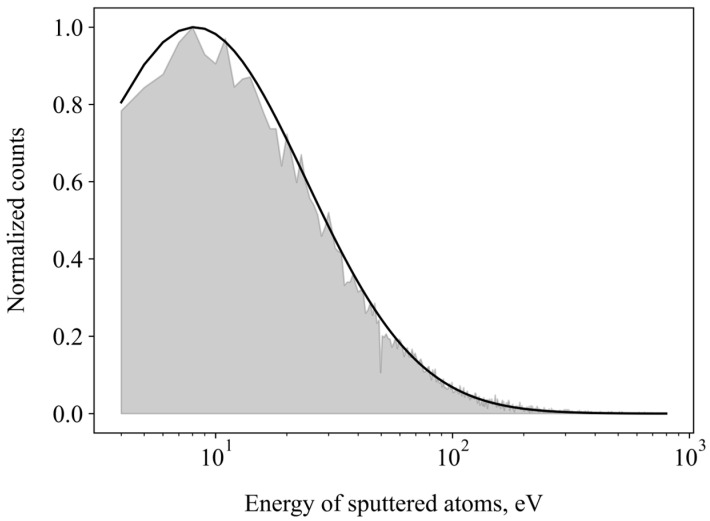
Energy profile of sputtered oxygen atoms. Black line is fit to the lognormal formula. Grey shaded area: normalized amount of sputtered oxygen atoms (yield) with a given energy.

The kinetic energies of all sputtered particles from various simulations were compiled into a histogram, referred to as an energy spectrum. This spectrum can be fitted to functions of surface binding energy. Figure 5 gives the integral energy spectrum of oxygen atoms sputtered for all energies and impact angles (grey filled area). To a good degree, it follows a lognormal distribution (black line). We can estimate the surface binding energy of WO_3_ from the following equation:

(2) $$Y\left(E_{sput}\right)\;\sim Ae^{\left(\frac{\ln\left(E\right)-E_{SB}}{\sigma }\right)}$$

*Y*(*E_sput_*) is the energy-dependent yield, *E* the energy of sputtered particles, and *E_SB_* the surface binding energy. The standard deviation *σ* and *A* are fitting parameters.

Using Equation (2), the effective surface binding energy of O on the WO_3_ surface is estimated to be 8.1 eV.

## 4. Conclusions

We have studied the events occurring upon the impact of Ar atoms on a WO_3_ surface under various irradiation conditions. We used classical molecular dynamics simulations to calculate large sets of trajectories. They were driven by a neural network potential energy function built from training data from density functional theory calculation. Analyzing the trajectories, we find a substantial effect of Ar incident energy on oxygen sputtering. At lower energies (100 eV), about 1% of the Ar atoms lead to the sputtering of 1–2 oxygen atoms, while at high energies (~800 eV), this ration changes to about 100%. Conversely, tungsten sputtering, even at 800 eV, remains very small, about 1% at 800 eV. This is in stark contrast to sputtering from pristine W surfaces, where for this energy a 1:1 ratio is observed. In summary, the analysis of angular probabilities showed that the angle of sputtered O atoms is largely unrelated to the one of the incoming Ar atoms. However, their impact angle has considerable influence on the sputtering yield. The largest one is observed at 60°. The energy spectrum of sputtered oxygen atoms follows a lognormal distribution to a good degree. This allowed us to estimate the effective O surface binding energy in WO_3_ to be approximately 8.1 eV.

## Figures and Tables

**Table 1:** Values of the fitted parameters A, a, B and b in Equation (1) for Ar impacts at perpendicular (0°) and 60° incidence angles.

Impact Angle	0°						60°					
Energy, eV	50	100	200	300	500	800	50	100	200	300	500	800
A	97	102	86	137	−0.84	1.52	94	217	308	296	278	1.38
a	0.82	1.10	1.13	1.43	4.34	1.45	1.31	0.98	0.98	1.07	1.16	0.82
B	97	102.33	86	137	−0.97	1.39	94	218	308	297	278	1.31
b	0.83	1.11	1.14	1.44	0.61	6.10	1.33	0.99	0.99	1.08	1.17	3.58

## Data Availability

No new data were created or analyzed in this study. Data sharing is not applicable to this article.

## Supplementary Materials

The following supporting information can be downloaded at: https://www.mdpi.com/article/10.3390/molecules29245928/s1, Figure S1: W-W potential energy curves; Figure S2: DFT and NNP calculated adsorption curve and work terms of surface Oxygen and W atoms; Table S1: Parameters of radial symmetry functions; Table S2: Parameters of angular symmetry functions; Table S3: Elastic constants of W; Table S4: Mean absolute error scores of the tested systems. Refs. [37,38] are cited in Supplementary Materials file.
