# Supplementary material for: Modelling the Impact of Argon Atoms on a WO3 Surface by Molecular Dynamics Simulations
Source: Molecules. 2024 Dec 16;29(24):5928. doi: 10.3390/molecules29245928 (PMC11678842; doi:10.3390/molecules29245928)
Supplement: Supplementary file 1 [file molecules-29-05928-s001.zip › molecules-3314550-supplementary.pdf]

Supplementary Materials

Modelling the impact of Argon atoms on a WO_3 Surface by Molecular dynamics simulations

Shokirbek Shermukhamedov[1,2,*], Thana Maihom[3] and Michael Probst[1,4,*]

[1] Institute of Ion Physics and Applied Physics, University of Innsbruck, Technikerstraße 25, 6020 Innsbruck, Austria
[2] Department of Chemistry, Ångström Laboratory, Uppsala University, 751 21 Uppsala, Sweden
[3] Division of Chemistry, Department of Physical and Material Sciences, Faculty of Liberal Arts and Science, Kasetsart University, Nakhon Pathom 73140, Thailand; t_maihom@hotmail.com
[4] Department of Materials Science and Engineering, School of Molecular Science and Engineering,
Vidyasirimedhi Institute of Science and Technology, Rayong 21201, Thailand
* Corresponding authors: michael.probst@uibk.ac.at; shokirbek.shermukhamedov@kemi.uu.se

S1. Symmetry functions

Radial and -angular Behler-type weighted atom-centered symmetry functions are defined in Eqns. (S1) and (S2) respectively, and are the same as in Ref.[1,2].

$$G_i^{rad} = \sum_{j \neq i} g(Z_j) e^{-\eta(R_{ij}-R_s)^2} f_c(R_{ij}) \tag{S1}$$

$$G_i^{ang} = 2^{1-\zeta} \sum_{i \neq j}^{N} \sum_{k \neq i,j}^{N} h(Z_j, Z_k)\left(1 + \lambda \cos\theta_{ijk}\right)^{\zeta} e^{-\eta(R_{ij}-R_s)^2} e^{-\eta(R_{ik}-R_s)^2} e^{-\eta(R_{jk}-R_s)^2} f_c(R_{ij}) f_c(R_{ik}) f_c(R_{jk}) \tag{S2}$$

Parameters of radial and angular symmetry functions employed to describe the local atomic environments in the input layer of the neural network for two elements (W and Ar) are given in Table S1.

Table S1: Parameters of radial symmetry functions employed to describe the local atomic environments in the input layer of the two elements neural network (W,O, Ar). r_{cut} is the cutoff radius and the meaning of the rest parameters refer to the definitions in articles[1,2].

η	R_S	r_{cut}
**1.35E+00**	0	1.30E+01
**1.35E+00**	6.190E-01	1.30E+01

**1.35E+00**	1.238E+00	1.30E+01
**1.35E+00**	1.857E+00	1.30E+01
**1.35E+00**	2.476E+00	1.30E+01
**1.35E+00**	3.095E+00	1.30E+01
**1.35E+00**	3.714E+00	1.30E+01
**1.35E+00**	4.333E+00	1.30E+01
**1.35E+00**	4.952E+00	1.30E+01
**1.35E+00**	5.571E+00	1.30E+01
**1.35E+00**	6.190E+00	1.30E+01
**1.35E+00**	6.810E+00	1.30E+01
**1.35E+00**	7.429E+00	1.30E+01
**1.35E+00**	8.048E+00	1.30E+01
**1.35E+00**	8.667E+00	1.30E+01
**1.35E+00**	9.286E+00	1.30E+01
**1.35E+00**	9.905E+00	1.30E+01
**1.35E+00**	1.052E+01	1.30E+01
**1.35E+00**	1.114E+01	1.30E+01
**1.35E+00**	1.176E+01	1.30E+01
**1.35E+00**	1.238E+01	1.30E+01
**1.35E+00**	1.300E+01	1.30E+01

Table S2: Parameters of angular symmetry functions employed to describe the local atomic environments in the input layer of the two elements neural network (W,O, Ar). r_{cut} is the cutoff radius. For the other parameters we refer to the definitions in articles[1,2].

$\boldsymbol{\eta}$	$\boldsymbol{\lambda}$	$\boldsymbol{\zeta}$	$\boldsymbol{r_{cut}}$	$\boldsymbol{R_S}$
**5.56E-02**	6.612E-02	-1	0	1.30E+01
**5.56E-02**	6.612E-02	1	0	1.30E+01
**5.56E-02**	6.612E-02	-1	0	1.30E+01
**5.56E-02**	6.612E-02	1	0	1.30E+01
**5.56E-02**	6.612E-02	-1	0	1.30E+01
**5.56E-02**	6.612E-02	1	0	1.30E+01
**5.56E-02**	6.612E-02	-1	0	1.30E+01
**5.56E-02**	1.00E+01	1	1.00E+00	1.30E+01
**5.56E-02**	1.30E+01	-1	1.00E+00	1.30E+01
**5.56E-02**	1.30E+01	1	1.00E+00	1.30E+01

S2. NNP validation

The lattice constant of bulk WO_3, calculated from the final neural network potential (NNP), was 3.84 Å (Figure S1). This value is within the range reported in previous DFT calculations but is slightly higher than the experimental values (Table S3). The observed discrepancies in lattice constants and bulk moduli may stem from the influence of the argon atoms and the surface structures included in the training dataset.

In addition, we evaluated static adsorption curves for argon and calculated the work functions for removing oxygen (two types) and tungsten atoms using both DFT and NNP. While the DFT-based results exhibit smooth curves, the NNP energy values show larger deviations from the reference data (Figure S2). These differences likely arise from the absence of specific configurations in the training dataset that correspond to the structures used in these tests. The NNP partially includes many-body terms in the sense of an "effective" potential energy curve. The corresponding atomic forces, however, show a strong linear correlation between the NNP and DFT predictions.

Table S4 presents the mean absolute error (MAE) scores for energy and forces across all tested systems. While the energy MAE for the adsorption curve is notably high at 14.32 meV/atoms, the MAE for the forces lower than in test set values – 1.31 eV/Å. This demonstrates that the NNP reproduces the forces, a critical factor when simulating irradiation by MD.

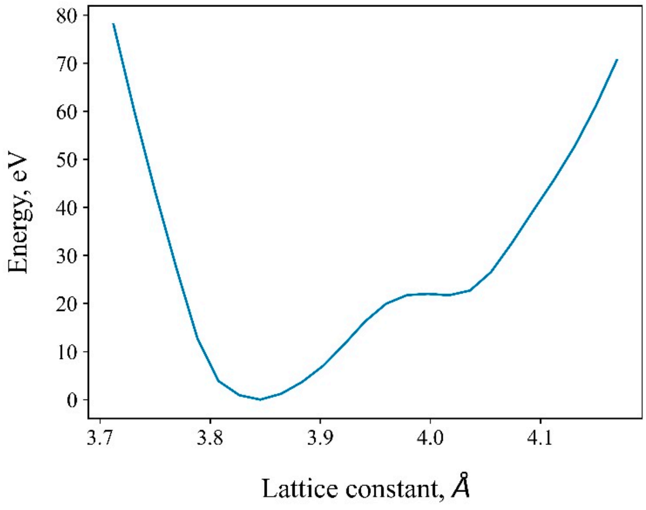

Figure S1. Interatomic W-W potential energy (a) and bcc-W lattice energy (b) curves calculated by using NNP.

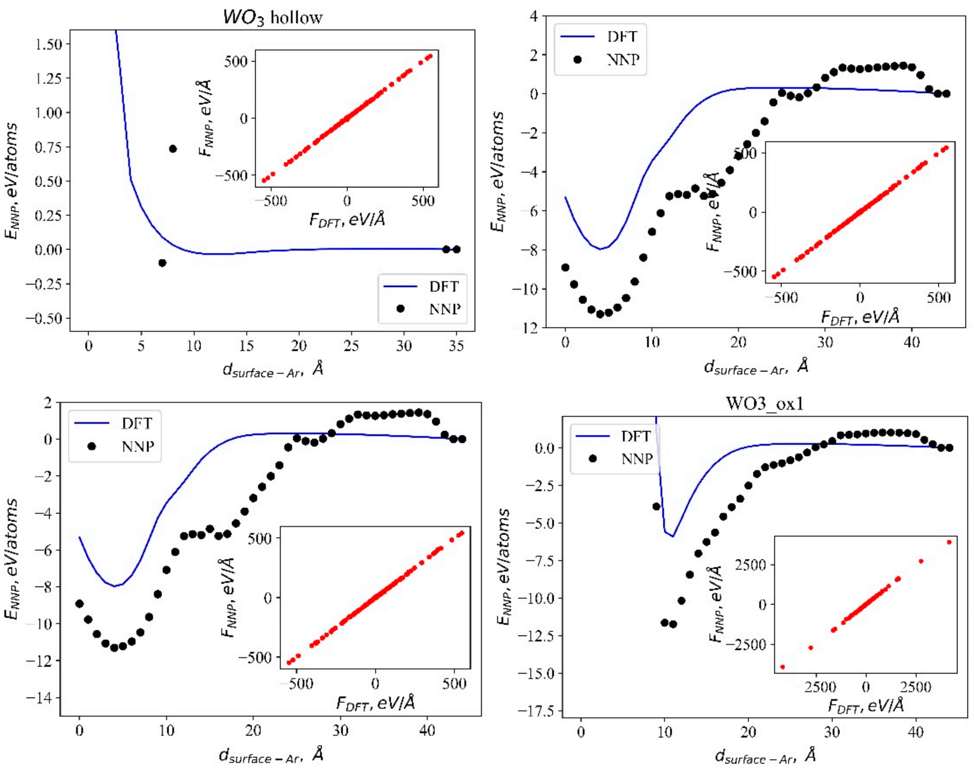

Figure S2. DFT and NNP calculated adsorption curve and work terms of surface Oxygen and W atoms.

Table S3. Elastic constants of WO_3 calculated in this work (using the data sets of previous calculations and experimental results.

	a (Å)	C_{11}	C_{12}	C_{44}	B
**LDA+U**[1]	3.82	546	35	71	205
**PBE-GGA**[2]	3.6	337.3	173.3	2.6	228
**LDA-Hedin−Lundqvist** [3]	3.76				257
**ABOP**[2]	3.698	225.8	171.4	334	225
**Expt.** [1]	3.71-3.75				
**Our work**	3.84	632	53	423	245

Table S4. Mean absolute error scores of the tested systems.

	**MAE**	
**System**	energy meV/atom	Forces eV/Å
**Ar - WO_3**	14.32	1.32
**W-WO_3**	0.62	1.42
**O (1) -WO_3**	37.06	1.31
**O (2) -WO_3**	60.52	1.37

References

1. Liu, X.; Fan, H. Electronic Structure , Elasticity , Debye Temperature and Anisotropy of Cubic WO 3 from First-Principles Calculation. R. Soc. open sci.Royal Soc. Open Sci. 2018, 5, 1–11.

2. Dendzik, Z.; Chrobak, D.; Nowak, R. Elastic Constants and Analytic Bond Order Potential for Atomistic Simulations of Simple Cubic Tungsten Trioxide. TASK Quarterly. Sci. Bull. Acad. Comput. Cent. Gdansk 2009, 13, 93–98.

3. Corà, F.; Stachiotti, M.G.; Catlow, C.R.A.; Rodriguez, C.O. Transition Metal Oxide Chemistry: Electronic Structure Study of WO3, ReO3, and NaWO3. J. Phys. Chem. B 1997, 101, 3945–3952, doi:10.1021/jp963724z.
